# Limited Clinical and Diagnostic Utility of Circulating Tumor DNA Detection in Patients with Early-Stage Well-Differentiated Thyroid Cancer: Comparison with Benign Thyroid Nodules and Healthy Individuals

**DOI:** 10.3390/healthcare9040386

**Published:** 2021-04-01

**Authors:** Yong Joon Suh, Mi Jung Kwon, Hye-Mi Noh, Hong Kyu Lee, Yong Joon Ra, Nan Young Kim

**Affiliations:** 1 Department of Breast and Endocrine Surgery, Hallym University Sacred Heart Hospital, Hallym University College of Medicine, 22, Gwanpyeong-ro 170 beon-gil, Dongan-gu, Anyang-si 14068, Gyeonggi-do, Korea; nicizm@hallym.or.kr; 2 Department of Pathology, Hallym University Sacred Heart Hospital, Hallym University College of Medicine, 22, Gwanpyeong-ro 170 beon-gil, Dongan-gu, Anyang-si 14068, Gyeonggi-do, Korea; 3 Department of Family Medicine, Hallym University Sacred Heart Hospital, Hallym University College of Medicine, 22, Gwanpyeong-ro 170 beon-gil, Dongan-gu, Anyang-si 14068, Gyeonggi-do, Korea; 4 Department of Thoracic and Cardiovascular Surgery, Hallym University Sacred Heart Hospital, Hallym University College of Medicine, 22, Gwanpyeong-ro 170 beon-gil, Dongan-gu, Anyang-si 14068, Gyeonggi-do, Korea; hklee0228@hallym.or.kr (H.K.L.); trimy@hallym.or.kr (Y.J.R.); 5 Hallym University Medical Center, Hallym Institute of Translational Genomics and Bioinformatics, Anyang 14068, Gyeonggi-do, Korea; honeyny@hallym.or.kr

**Keywords:** circulating tumor DNA, thyroid cancer, mutation, clinical diagnosis

## Abstract

Limited data are available on the diagnostic utility of circulating tumor DNA (ctDNA) in early-stage thyroid cancers for *BRAF*, *KRAS*, *NRAS*, and *TERT* promoter mutations, which are known detectable markers for thyroid cancers. Here, we analyzed the above driver mutations in ctDNA and matched neoplastic tissues from patients with early-stage thyroid cancers in order to investigate diagnostic utility of circulating markers in distinguishing from other mimicking thyroid lesions and healthy individuals. In total, 73 matched neoplastic tissue and plasma samples [thyroid cancers (n = 62), benign thyroid disorders (n = 8), and parathyroid lesions (n = 3)] and 54 plasma samples from healthy individuals (as controls) were analyzed for *BRAF*, *KRAS*, *NRAS*, and *TERT* promoter mutations using peptide nucleic acid clamp real-time PCR. Although only one patient with an indeterminate lesion on thyroid cytology showed *KRAS* mutation (codon 146) in the preoperative plasma, that *KRAS* mutation was not identified in the stage I papillary thyroid carcinoma tissue. In the remaining 72 plasma samples, no other mutations were identified in *BRAF*, *NRAS*, and *TERT* promoter genes. The concordance rates of mutational results between the plasma and tumor tissue or metastatic lymph node were very low. One (1.9%) of the 54 healthy individuals harbored a *KRAS* mutation in the plasma samples. The ctDNA results did not represent the mutational profile of primary or metastatic thyroid cancers, warranting a caution for interpretation. The clinical utility of *BRAF*, *KRAS*, *NRAS*, and *TERT* promoter mutation analysis on ctDNA appears to be limited to early-stage thyroid cancers.

## 1. Introduction

Thyroid cancer has been one of the most widespread malignancies of the endocrine-related system over the past few decades, with a vivid increasing rate [1,2]. In the Korean population, there has been a recent rise in the incidence of thyroid cancers that represent the fourth most common cancers and the second most common malignancies in Korean women, raising nationwide concern [1]. Papillary thyroid carcinoma (PTC) and follicular thyroid carcinoma (FTC) are the most common types of thyroid malignancies, accounting for more than 90% [2]. Ultrasound-guided fine-needle aspiration biopsy (FNAB) is the most reliable nonsurgical test for the detection of thyroid cancer, representing the gold standard in the management of thyroid nodules, allowing the distinction between lesions that need surgery and those that do not [3]. Despite the high sensitivity and specificity of thyroid cytology, indeterminate or non-diagnostic results in 20–40% of FNAB limit its utility and may complicate the management of thyroid nodules [3,4]. Addition of *BRAF* V600E mutation analysis in FNAB has been employed in an attempt to further refine diagnostic accuracy in the case of indeterminate Bethesda III–IV lesions [5], which is encouraging but does not fully address the problem of diagnosis of indeterminate lesions [6]. Therefore, development of reliable molecular markers for these tumors through a less invasive technique than FNAB is significant in terms of diagnosis, prognosis, treatment, and follow-up [7]. 

Genetic and epigenetic alteration of the RAS–RAF–MEK–MAPK–ERK pathway, and mutations in *NRAS*, *KRAS*, and *BRAF*, have been shown to strongly influence the development of thyroid cancers [7,8,9]. *TERT* promoter mutation, which induces aberrant activation of telomerase, has also been reported as a risk factor for disease progression of PTC and transformation to anaplastic carcinoma [10]. Circulating tumor DNA (ctDNA) testing in plasma, evaluated in several cancer models, exhibits the potential to be a supplemental tool for conventional tumor biopsies or cytology [11,12], particularly for advanced metastatic tumors or unresectable cancer [13,14]. Because ctDNA is composed of small fragments of nucleic acid that are released from apoptotic or necrotic tumor cells and circulate in the blood [11], sampling ctDNA from blood may potentially allow early diagnosis of thyroid cancer. Nevertheless, there have been conflicting results regarding early detection and tumor recurrence using ctDNA as a diagnostic marker in the plasma of patients with PTC [13,15,16,17,18]. It must be noted that most studies of ctDNA in thyroid cancer have focused on patients in late stages of the disease or otherwise the specific description for the stages were unclear [15,16,17,18,19]. Limited data are available on the evaluation of ctDNA detection in early-stage thyroid cancer, which was only a series of overall thyroid cancers regardless of the stages [19]. Thus, detection of genomic abnormalities in ctDNA and their association with clinical characteristics in early-stage thyroid cancer need to be clarified. 

Here, we comprehensively analyzed *BRAF*, *NRAS*, *KRAS*, and *TERT* promoter mutations from 73 matched neoplastic tissue and plasma DNA samples, including preoperative plasma, along with 27 matched metastatic lymph nodes, in order to investigate diagnostic utility of circulating markers in distinguishing early-stage thyroid cancers from other mimicking thyroid lesions and healthy individuals. 

## 2. Materials and Methods

### 2.1. Patient Population and Blood Sample Collection

This prospective study involved 73 consecutive intrathyroid neoplasmic patients undergoing initial curative surgery and control healthy individuals (n = 54) between September 2018 and May 2019. Patients with thyroid cancers (n = 62), benign thyroid disorders (n = 8), and parathyroid lesions (n = 3) underwent initial curative surgery after cytological diagnosis based on ultrasound imaging and sono-guided aspiration. Finally, 73 matched tumor and plasma DNA samples from patients with thyroid neoplasms (pathologically proven: 60 PTCs, 2 FTCs, 6 nodular hyperplasias, 2 Graves’ diseases, 1 parathyroid carcinoma, 1 parathyroid adenoma, and 1 parathyroid hyperplasia) were investigated. Additionally, 27 cases with metastatic nodes were investigated. Healthy controls (n = 54) were individuals who visited the hospital for routine physical examination. All subjects provided written informed consent for the procedure, and the study protocol was approved by the Institutional Review Board of Hallym University Sacred Heart Hospital (IRB No. HALLYM 2018-07-018). Peripheral blood samples (10 mL) were obtained from each patient before surgery or from each healthy individual during examination for subsequent analyses.

### 2.2. DNA Extraction 

DNA was extracted from 4 μm thick sections of 10% neutral formalin-fixed paraffin embedded (FFPE) tumor tissue blocks using a Maxwell^®^ 16 FFPE Tissue LEV Purification Kit for DNA (Promega, Leiden, The Netherlands) according to the manufacturer’s instructions. The sections were deparaffinized in xylene and washed in ethanol prior to DNA extraction. 

For plasma DNA, 10 mL peripheral blood samples collected in PAXgene Blood ccfDNA Tubes (PreAnalytiX GmbH, Hombrechtikon, Switzerland), transported within 1 h to the laboratory, were centrifuged twice at 1600 and 14,000 rpm for 10 min at 4 °C. Plasma was aliquoted into 1.5 mL tubes after centrifugation and stored at −80 °C until genetic analysis. Isolation of cell-free DNA from plasma was carried out using an OptiPure cfDNA kit (TAN Bead, Taoyuan City, Taiwan) as per the manufacturer’s instructions.

After the DNA was eluted in 50 μL of elution buffer, its concentration and purity were measured using a NanoDrop ND-1000 spectrophotometer (NanoDrop Technologies, Wilmington, DE, USA). The extracted DNA was stored at −20 °C until further experiments.

### 2.3. BRAF, NRAS, KRAS, and TERT Promoter Gene Analysis

A total of 91 activating mutations in *BRAF* (codon 600), *NRAS* (codon 12, codon 13, codon 59, codon 61, codon 117, and codon 146), *KRAS* (codon 12, codon 13, codon 59, codon 61, codon 117, and codon 146), and *TERT* promoter (C250 and C228) genes were investigated. *BRAF*, *NRAS*, *KRAS*, and *TERT* promoter gene mutations were detected by performing peptide nucleic acid (PNA)-mediated real-time polymerase chain reaction (PCR) clamping with PNAClamp™ *BRAF* Mutation Detection Kit, PNAClamp™ *KRAS* Kit, PNAClamp™ *NRAS* Kit, and PNAClamp™ *TERT* Mutation Detection Kit (PANAGENE, Daejeon, Korea), respectively, according to the manufacturer’s instructions, and as described previously [15,20]. PNA is a synthetic DNA analog in which the phosphodiester backbone is replaced by a peptide-like repeat formed by (2-aminoethyl)-glycine units. This kit is an amplified DNA test for qualitative detection using PNA probes and Ct analysis in a real-time PCR system. The principle underlying the PNAClamp™ technology is that PNA inhibits the amplification of wild-type DNA by hybridizing to wild-type sequences, so that the mutant DNA is predominantly amplified. It is then detected using a DNA-intercalating dye.

PCR amplification for *BRAF*, *KRAS*, and *NRAS* was performed in a total volume of 20 μL that contained 7 μL of DNA, 3 μL of respective PNA mix, and 10 μL of 2× premix, in a total volume of 20 μL, whereas that of *TERT* promoter consisted of 5 μL of DNA and 15 μL of PNA mix. 

The DNA in the reaction was then amplified using the CFX96 real-time PCR system (Bio-Rad, Hercules, CA, USA) with the following thermal programs: for *BRAF*, pre-denaturation at 94 °C for 5 min, followed by 40 cycles of four temperature steps: 94 °C for 30 s, 70 °C for 20 s, 63 °C for 30 s, and 72 °C for 30 s, followed by a final extension at 72 °C for 5 min; for *KRAS* and *NRAS*, two holding periods of 50 °C for 2 min and 95 °C for 15 min, 15 cycles of denaturation at 95 °C for 30 s, annealing at 70 °C for 20 s, and extension at 63 °C for 60 s, 35 cycles of denaturation at 95 °C for 10 s, annealing at 53 °C for 20 s, and extension at 73 °C for 20 s, and a melting curve step with increase in temperature from 35 °C to 75 °C; for *TERT* promoter, pre-denaturation at 95 °C for 15 min, followed by 40 cycles of four temperature steps: 95 °C for 30 s, 80 °C for 20 s, 76 °C for 20 s, 72 °C for 20 s, and 68 °C for 30 s, followed by a final extension of 72 °C for 30 s.

The threshold cycle (Ct) was automatically calculated from the PCR amplification plots in which fluorescence was plotted against the number of cycles. Delta-Ct values (∆Ct) were calculated as the Ct value of the PCR with the PNA control minus the Ct value of the PCR of the samples. Higher ∆Ct means that the mutant was efficiently amplified. Each cutoff value was used for determining the presence of mutant DNA.

### 2.4. Statistical Analysis

Demographic data are presented as the mean ± SD, or as number (n) and percentage. Statistical analyses of *BRAF* mutation and clinicopathological features in PTCs were performed using the Chi-square test or the 2-tailed Fisher’s exact test. Concordance between mutation analysis of archival tumor tissue and mutation analysis of cell-free DNA from plasma samples was calculated using a kappa coefficient. The SPSS statistical software (ver. 20) (IBM Corp., Armonk, NY, USA) was used for all statistical analyses. *p* values < 0.05 were considered to be statistically significant. 

## 3. Results

**Figure 1:**
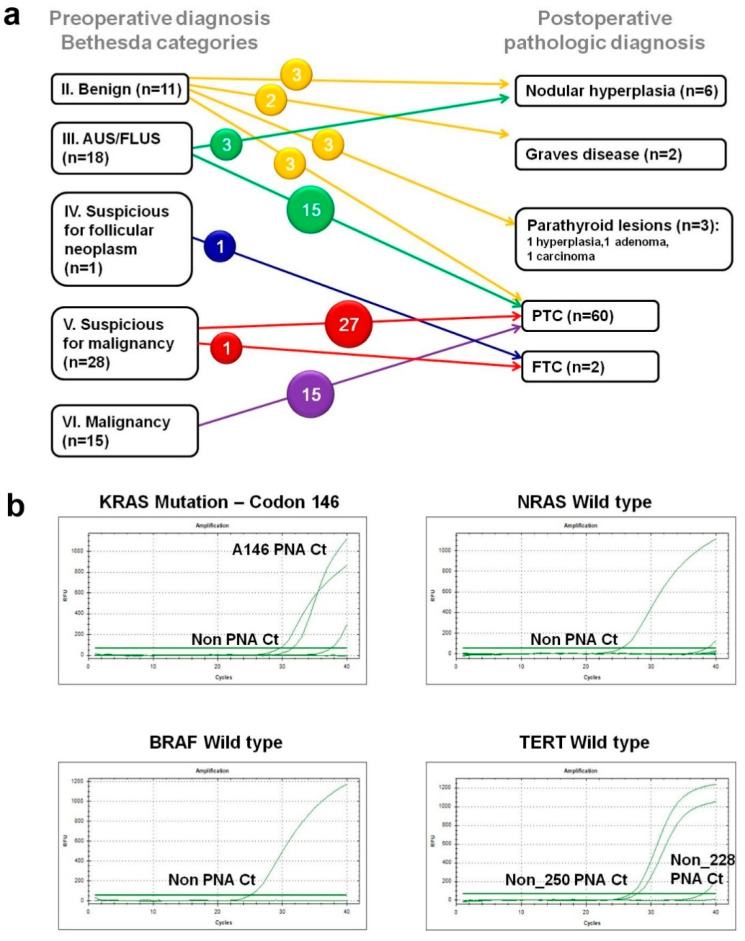
(**a**) Postoperative final diagnostic diagrams from preoperative diagnosis of fine-needle aspiration according to the Bethesda categories. Only Bethesda category III (indeterminate lesions) shows cell-free DNA of *KRAS* mutation in the preoperative plasma sample. (**b**) Representative images of peptide nucleic acid-mediated real-time PCR clamping analysis in the 127 plasma samples, indicating only one *KRAS* mutation at codon 146, and others indicating wild-type *NRAS* gene, wild-type *BRAF* gene, and wild-type *TERT* promoter gene.

Baseline characteristics of the study population and tumor status are summarized in Table 1. Preoperative cytologic results of Bethesda categorization for the 73 nodules and their final pathologic diagnosis after surgery are summarized in Figure 1a. Among them, only one patient, preoperatively diagnosed with Bethesda III (atypia of undetermined significance) in the thyroid cytology, showed one *KRAS* mutation at codon 146 in the preoperative plasma sample (Figure 1b). That 53-year-old female patient was diagnosed with stage I (pT3bN0aM0) PTC, and the tissue contained 3 foci of PTCs with microscopic perithyroid soft tissue invasion in both thyroid lobes, measuring 0.3 × 0.2 cm (right upper pole), 1.1 × 0.7 cm (right mid to lower pole), and 0.7 × 0.3 cm (left mid portion). However, this *KRAS* mutation was not identified in the tumor tissue. Whole-body ^18^F-FDG PET/CT scan was performed for staging the extent of PTC and to rule out other primary malignancies, which did not show any increased FDG uptake, suggestive of no suspected malignancies. However, in the other 72 plasma samples, no other mutations were identified in *BRAF*, *NRAS*, *KRAS*, and *TERT* promoter genes. As a result, no significant differences in ctDNAs were detected in any of the remaining 72 patients (Table 2). 

However, one *KRAS* mutation at codon 146 was observed in one person, a 43-year-old male, out of the 54 healthy individuals. The past medical history of that individual was non-specific. Thus, ctDNA analyses of *BRAF*, *NRAS*, *KRAS*, and *TERT* promoter gene mutations could not differentiate between samples from benign and malignant thyroid lesions or healthy individuals. 

Among 60 PTCs, 41 primary tumor tissues (68.3%) harbored the *BRAF* mutation, while 2 (3.5%) harbored *TERT* promoter mutation. However, none of these cases who were positive for either *BRAF* mutation or *TERT* promoter mutation in primary tumors had detectable *BRAF* or *TERT* promoter mutation in the corresponded plasma. There were no significant differences in clinical or pathologic characteristics between the PTC patients with and without *BRAF* mutation (Table 3).

It was observed that 27 out of 60 PTCs had the matched metastatic lymph nodes and the plasma samples (Table 4). In those 27 metastatic node tissues of PTC, 17 (63.0%, 17/27), 3 (11.1%, 3/27), and 1 (3.7%, 1/27) metastatic nodal tissues showed *BRAF*, *NRAS*, and *TERT* promoter mutations, respectively. However, none of the patients who tested positive for either *BRAF* mutation, *NRAS* mutation, or *TERT* promoter mutation in primary tumors had detectable mutations in their plasma.

Taken together, the concordance rates of the tumor tissue versus plasma and the metastatic lymph node versus plasma of *BRAF* results were 23.9% (17/71) and 11.1% (3/27), respectively, which did not display any statistical association (κ = 0.032, *p* = 0.465; κ = 0.030, *p* = 0.561, respectively). The concordance rates of the tumor tissue or the metastatic lymph node versus plasma of *KRAS* results were 78.9% (56/71) and 70.4% (19/27), respectively, which were not statistically significant (κ = 0.059, *p* = 0.410; κ = 0.033, *p* = 0.547, respectively). The concordance rates of the tumor tissue or the metastatic lymph node versus plasma of *NRAS* results were 74.3% (52/70) and 55.6% (15/27), respectively, which were not statistically significant (κ = 0.049, *p* = 0.511; κ = 0.061, *p* = 0.332, respectively). The concordance rates of the tumor tissue or the metastatic lymph node versus plasma of *TERT* promoter results were 84.3% (59/70) and 85.2% (23/27), respectively, which did not show statistical significance (κ = 0.076, *p* = 0.397; κ = 0.029, *p* = 0.719, respectively) either.

## 4. Discussion

In this study, 73 matched neoplastic tissue and plasma DNA samples and 54 plasma DNA samples from healthy individuals were examined for the diagnostic utility of ctDNA in thyroid cancers focusing on *BRAF*, *KRAS*, *NRAS*, and *TERT* promoter mutations. The ctDNA analyses of *BRAF*, *NRAS*, *KRAS*, and *TERT* promoter gene mutations could not differentiate between patients with benign or malignant thyroid lesions and healthy individuals. The statistically significant concordance with respect to mutational results between tumor tissues, metastatic nodal tissues, and matched plasma was not observed in early-stage thyroid cancers. 

In the present study, of 60 PTCs, 41 were *BRAF*-mutated (68.3%) and *TERT* promoter-mutated (3.5%), in contrast to no *NRAS* or *KRAS* mutation in the tumor tissues. In addition, the *BRAF* V600E mutation was absent in plasma or tissue DNA samples obtained from patients with benign follicular adenomas or adenomatous goiter, which is consistent with an earlier report [19]. Furthermore, frequencies of *BRAF* and *TERT* promoter mutations in the tumor tissues were also found to be in line with previous studies [10,21]. The frequency of the *BRAF* V600E mutation in PTC ranged from 29% to 83% in different regions and from 70% to 80% in Korea, a *BRAF* V600E mutation-prevalent area [9]. The *TERT* promoter mutation has been identified in ~10% of PTCs and is closely associated with aggressive clinical behavior of PTCs [10,21]. However, the corresponding ctDNAs of *BRAF*, *NRAS*, and *TERT* promoter mutations were all negative. We found only one *KRAS* mutation in the plasma from preoperative indeterminate cytology, and postoperative pathologically proven stage I PTC. However, that *KRAS* mutation was not identified in the tumor tissue. Whole-body ^18^F-FDG PET/CT scan did not reveal any other suspected malignancy. The *KRAS* G12V mutation detected via ctDNA analysis has been reported in the plasma of 1 patient with stage IVA PTC, with ctDNA being the only mutation (1.8%) detected in that patient [13]. This *KRAS* mutation was detected in both plasma and tumor tissue [13], which was in contrast to the present study. In order to address the possibility of *KRAS* mutation being detected only in the circulating plasma DNA and not in the tumor tissue, our findings agree with the previous studies, where the discordant result of primary tissue (wild-type) and plasma (mutant) has been also described in colorectal cancers [22] and pancreatic cancers [23]. It is possible that the discordance between primary tumor and circulating tumor cells accounts for the tumors being heterogeneous, harboring small subsets of cells with specific mutations not detected in routine diagnostics [23]. Future studies are warranted to determine clinical significance of discordant serum *KRAS* mutation through larger prospective studies of PTCs. Despite the highly sensitive technique being used in our study, circulating *BRAF*, *NRAS*, and *TERT* promoter mutation alleles were not detected in the plasma of patients with PTC and FTC, with the exception of one *KRAS* mutation in the patient with indeterminate lesion. Very low detectable or undetectable ctDNA in the thyroid cancers has also been raised as an issue by previous studies [13,18]. These low rates of detection may be related to the fact that the majority of thyroid cancers are PTCs with a propensity for lymphatic spread rather than hematogenous metastasis [24,25]. Furthermore, it is consistent with previous data showing that, unlike other cancers detectable more than 75%, such as advanced pancreatic, ovarian, colorectal, bladder, gastroesophageal, breast, melanoma, hepatocellular, and head and neck cancers, less than 50% of patients even with metastatic thyroid cancer harbored detectable ctDNA [14]. A clinical trial of ctDNA in patients with thyroid nodules was also terminated due to preliminary results indicating that this test had a significant number of false-negative results (NCT02778412). The clinical implication of ctDNA seems to be dependent on the tumor type and stage. Therefore, our results raise the question about the clinical utility of *BRAF*, *KRAS*, *NRAS*, and *TERT* promoter mutation analysis using ctDNAs of patients with thyroid cancers.

Using a relatively large number of healthy subjects to detect cancer-associated mutations in liquid biopsy, our study leads to a cautionary note that identification of driver mutations may not signify presence of disease. One (1.9%) of the 54 healthy individuals harbored a *KRAS* mutation, as detected in the plasma samples. Similarly, *KRAS* mutation (2.6%) independent of tumor tissue has been reported previously in healthy subjects [26]. One study has reported the presence of ctDNA, including *NRAS* mutation, *TP53* mutation, *GNAS* mutation, and both *KRAS* and *TP53* mutations, in 4% of healthy individuals, with the results indicating the false-positive rate of this method [17]. Gormally et al. [27] found that 3% of the control population had *TP53* mutations, and 1% had *KRAS* mutations, none of whom developed cancer. The study considered the mutational results detected in a high cancer-risk population as positive results, rather than a low cancer-risk population [27]. Because circulating free DNA may be derived from apoptotic or necrotic cells and can be released actively from normal and diseased cells, the detection of mutations in a background of normal circulating free DNA molecules may not be indicative of tumoral origin.

Lymph node metastasis is frequent in PTC, as seen in 20–90% of patients, and is associated with locoregional recurrence [24,25]. Considering tumor heterogeneity, analysis of ctDNA could theoretically provide more comprehensive and representative information regarding metastasis of multiple tumor deposits [28]. Using 27 matched metastatic lymph node and plasma samples from 60 patients with PTC, *BRAF* mutations (63.0%), *NRAS* mutations (11.1%), and *TERT* promoter mutations (3.7%) were detected in metastatic nodal tissues. However, we found no statistically significant concordance in any *BRAF*, *KRAS*, *NRAS*, and *TERT* promoter mutation between the tumor tissue versus plasma, and the metastatic lymph node versus plasma. 

The limitation of the current study may be that we failed to prove the clinical utility of *BRAF*, *KRAS*, *NRAS*, and *TERT* promoter mutation as circulating markers in early-staged thyroid cancers distinguishing from benign thyroid lesions or healthy individuals. It might be that larger amounts of blood may be needed to capture ctDNA-carrying mutations. We could not additionally analyze the ctDNA results by other methods including droplet digital polymerase chain reaction (ddPCR) or next-generation sequencing to validate, due to lack of remaining plasma samples [29]. The reason for the higher failed rate in plasma *BRAF* mutational analysis than others in the study may be in the order of repetition of freezing and thawing extracted DNA samples from *TERT*, *KRAS*, *NRAS* to *BRAF*, presumably leading to gradually decreased DNA quality. Chan et al. [30] reported that repeated freezing and thawing of plasma or extracted DNA would affect the integrity of plasma DNA. Given that ctDNA shows little promise for this patient group, other options such as different liquid biopsy approaches might be worth investigating for their diagnostic utility in this cancer for future research [31]. 

## 5. Conclusions

The detection of ctDNA from plasma did not represent the mutation profiles of primary or metastatic thyroid cancers, warranting a caution in the interpretation. The use of ctDNA to assist cytology in cases of indeterminate lesions was not helpful in improving the diagnoses of thyroid disorders, which seems to be limited still in routine clinical practice.

## Figures and Tables

**Table 1:** Clinical characteristics of patients.

Characteristics of All Subjects Enrolled in the Study	N = 127 (%)
Final pathology of neoplasms (n = 73)	
Thyroid cancers	62 (48.8)
Papillary carcinoma	60
Follicular carcinoma	2
Nodular hyperplasia	6 (4.7)
Graves’ disease	2 (1.6)
Parathyroid neoplasm	3 (3.6)
Parathyroid carcinoma	1
Parathyroid adenoma	1
Parathyroid hyperplasia	1
Healthy individual	54 (65.9)
Characteristics of patients with thyroid cancers	n = 62 (%)
Gender (n = 62)	
Male	18 (29.0)
Female	44 (71.0)
Age (y), median (n = 62)	56 (range, 21–85)
<55	38 (61.3)
≥55	24 (38.7)
Tumor resection procedure (n = 62)	
Hemithyroidectomy	23 (37.1)
Total thyroidectomy	39 (62.9)
Neck dissection (n = 62)	
Central neck dissection	52 (83.9)
Central neck dissection + MRND	10 (16.1)
Tumor size, mean ± SD (cm) (n = 62)	1.3 ± 1.6 (range, 0.3–10.5)
≤1cm	39 (62.9)
>1cm	23 (37.1)
T category (n = 62)	
T1	53 (85.5)
T2	2 (3.2)
T3a	2 (3.2)
T3b	5 (8.1)
N category (n = 62)	
N0	35 (56.5)
N1	27 (43.5)
AJCC stage (n = 62)	
Stage I	54 (87.1)
Stage II	8 (12.9)
Resection margin status (n = 62)	
R0	60 (96.8)
R1	2 (3.2)
Multiplicity (n = 62)	
Yes (≥2)	21 (33.9)
No	41 (66.1)
Extrathyroid extension (n = 62)	
No	35 (56.5)
Microscopic	18 (29.0)
Gross	9 (14.5)
Lymphovascular invasion (n = 62)	
Positive	23 (37.1)
Negative	39 (62.9)
Hashimoto’s thyroiditis (n = 62)	
Absent	54 (87.1)
Present	8 (12.9)

Abbreviations: MRND, modified radical neck dissection; SD, standard deviation; AJCC, American Joint Committee on Cancer.

**Table 2:** Mutation results of patients with thyroid cancers, benign thyroid or parathyroid lesions, and healthy individuals.

	*BRAF* (%)		*NRAS* (%)		*TERTp* (%)		*KRAS* (%)	
	MT	WT	MT	WT	MT	WT	MT	WT
Thyroid ca								
Primary (n = 62)	41 (66.1)	19 (30.6)	0 (0)	59 (95.2)	3 (4.8)	56 (90.2)	0 (0)	60 (96.8)
Meta LN (n = 27)	17 (63.0)	10 (37.0)	3 (11.1)	24 (88.9)	1 (3.7)	26 (96.3)	1 (3.7)	26 (96.3)
Plasma (n = 62)	0 (0)	30 (48.4)	0 (0)	46 (74.2)	0 (0)	56 (90.3)	1 (1.6)	50 (80.6)
NH (n = 6)	0 (0)	5 (83.3)	0 (0)	3 (50)	0 (0)	4 (66.7)	0 (0)	4 (66.7)
Graves’ ds (n = 2)	0 (0)	2 (100)	0 (0)	2 (100)	0 (0)	2 (100)	0 (0)	2 (100)
Parathyroid lesion (n = 3)	0 (0)	0 (0)	0 (0)	3 (100)	0 (0)	3 (100)	0 (0)	3 (100)
Healthy individuals (n = 54)	0 (0)	19 (35.2)	0 (0)	25 (46.3)	0 (0)	49 (90.7)	1 (1.9)	39 (72.2)

Abbreviations: MT, mutated; WT, wild-type; ca, carcinoma; LN, lymph node; NH, nodular hyperplasia; ds, disease.

**Table 3:** Clinical and pathological characteristics of *BRAF* mutation in papillary thyroid carcinoma.

	*BRAF*			*p*
	Total	Mutated	Wild-Type	
	n = 58	n = 41(70.7%)	n = 17(29.3%)	
Sex				0.519
Male	17 (29.3)	11 (26.8)	6 (35.3)	
Female	41 (70.7)	30 (73.2)	11 (64.7)	
Age (y)				0.743
<55	36 (62.1)	26 (63.4)	10 (58.8)	
≥55	22 (37.9)	15 (36.6)	7 (41.2)	
Tumor size				0.129
≤1 cm	38 (65.5)	24 (58.5)	14 (82.4)	
>1 cm	20 (34.5)	17 (41.5)	3 (17.6)	
pT category				0.583
pT1-2	53 (91.4)	38 (92.7)	15 (88.2)	
pT3	5 (8.6)	3 (7.3)	2 (11.8)	
Nodal status				0.397
pN0	32 (55.2)	21 (51.2)	11 (64.7)	
pN1	26 (44.8)	20 (48.8)	6 (35.3)	
AJCC stage				0.401
I	51 (87.9)	37 (90.2)	14 (82.4)	
II	7 (12.1)	4 (9.8)	3 (17.6)	
Multiplicity				0.791
Yes (≥2)	19 (32.8)	13 (31.7)	6 (35.3)	
No	39 (67.2)	28 (68.3)	11 (64.7)	
Extrathyroid extension				1.000
Microscopic/absent	51 (87.9)	36 (87.8)	15 (88.2)	
Gross	7 (12.1)	5 (12.2)	2 (11.8)	
Lymphovascular invasion				0.764
Positive	20 (34.5)	15 (36.6)	5 (29.4)	
Negative	38 (65.5)	26 (63.4)	12 (70.6)	
Hashimoto’s thyroiditis				1.000
Absent	51 (87.9)	36 (87.8)	15 (88.2)	
Present	7 (12.1)	5 (12.2)	2 (11.8)	

Abbreviations: AJCC, American Joint Committee on Cancer.

**Table 4:** Comparisons of *BRAF*, *KRAS*, *NRAS*, and *TERT* promoter mutation results among the primary tumor tissue, metastatic nodal tissue, and plasma in 27 PTCs with metastatic lymph nodes.

Case No.	*BRAF*			*KRAS*			*NRAS*			*TERTp*		
	Tumor	Meta LN	Plasma	Tumor	Meta LN	Plasma	Tumor	Meta LN	Plasma	Tumor	Meta LN	Plasma
**Concordant**												
#1	V600	V600	WT	WT	WT	WT	WT	WT	WT	WT	WT	WT
#6	V600	V600	failed	WT	WT	failed	WT	WT	failed	WT	WT	WT
#7	V600	V600	failed	WT	WT	WT	WT	WT	failed	WT	WT	WT
#9	V600	V600	failed	WT	WT	WT	WT	WT	WT	WT	WT	WT
#11	V600	V600	failed	WT	WT	WT	WT	WT	WT	WT	WT	WT
#14	V600	V600	WT	WT	WT	WT	WT	WT	failed	WT	WT	WT
#15	V600	V600	WT	WT	WT	WT	WT	WT	WT	WT	WT	WT
#16	V600	V600	WT	WT	WT	WT	WT	Codon13	WT	WT	WT	WT
#17	V600	V600	WT	WT	WT	WT	WT	WT	WT	WT	WT	WT
#20	V600	V600	WT	WT	WT	failed	WT	Codon61	WT	WT	WT	failed
#21	V600	V600	WT	WT	WT	failed	WT	WT	WT	WT	WT	failed
#23	V600	V600	WT	WT	WT	failed	WT	WT	failed	C228	WT	WT
#24	V600	V600	failed	WT	WT	WT	WT	WT	WT	WT	WT	WT
#26	V600	V600	failed	WT	WT	WT	WT	WT	WT	WT	WT	WT
#27	V600	V600	failed	WT	WT	WT	WT	WT	WT	WT	WT	WT
#5	WT	WT	failed	WT	WT	WT	failed	WT	failed	failed	WT	WT
#12	WT	WT	failed	WT	WT	WT	WT	WT	failed	WT	WT	WT
#18	WT	WT	WT	WT	WT	WT	WT	WT	WT	WT	WT	WT
#25	WT	WT	failed	WT	WT	WT	WT	WT	WT	WT	WT	WT
**Discordant**												
#2	V600	WT	failed	WT	WT	failed	WT	WT	failed	WT	WT	WT
#10	V600	WT	failed	WT	WT	WT	WT	WT	WT	WT	WT	WT
#13	V600	WT	failed	WT	WT	WT	WT	Codon61	failed	WT	WT	WT
#19	V600	WT	WT	WT	WT	failed	WT	WT	WT	WT	WT	failed
#22	V600	WT	WT	WT	WT	WT	WT	WT	WT	WT	WT	WT
#3	WT	V600	failed	WT	WT	WT	WT	WT	WT	WT	C250	WT
#4	WT	V600	WT	WT	WT	WT	WT	WT	WT	WT	WT	WT
#8	failed	WT	failed	failed	WT	WT	WT	WT	failed	failed	WT	WT

Abbreviations: LN, lymph node; WT, wild-type; MT, mutated.

## Data Availability

The data used to support the findings of this study are available from the corresponding author upon request.

## References

[1] Jung K.W., Won Y.J., Kong H.J., Lee E.S. (2019). Prediction of Cancer Incidence and Mortality in Korea, 2019. Cancer Res. Treat..

[2] Conzo G., Avenia N., Bellastella G., Candela G., de Bellis A., Esposito K., Pasquali D., Polistena A., Santini L., Sinisi A.A. (2014). The role of surgery in the current management of differentiated thyroid cancer. Endocrine.

[3] Alexander E.K., Kennedy G.C., Baloch Z.W., Cibas E.S., Chudova D., Diggans J., Friedman L., Kloos R.T., LiVolsi V.A., Mandel S.J. (2012). Preoperative diagnosis of benign thyroid nodules with indeterminate cytology. N. Engl. J. Med..

[4] Cibas E.S., Ali S.Z. (2017). The 2017 Bethesda System for Reporting Thyroid Cytopathology. Thyroid.

[5] Cohen Y., Rosenbaum E., Clark D.P., Zeiger M.A., Umbricht C.B., Tufano R.P., Sidransky D., Westra W.H. (2004). Mutational analysis of BRAF in fine needle aspiration biopsies of the thyroid: A potential application for the preoperative assessment of thyroid nodules. Clin. Cancer Res..

[6] Su X., Jiang X., Xu X., Wang W., Teng X., Shao A., Teng L. (2016). Diagnostic value of BRAF (V600E)-mutation analysis in fine-needle aspiration of thyroid nodules: A meta-analysis. Onco Targets Ther..

[7] Nikiforov Y.E., Nikiforova M.N. (2011). Molecular genetics and diagnosis of thyroid cancer. Nat. Rev. Endocrinol..

[8] Chiosea S., Asa S.L., Berman M.A., Carty S.E., Currence L., Hodak S., Nikiforov Y.E., Richardson M.S., Seethala R.R., Sholl L.M. (2017). Template for Reporting Results of Biomarker Testing of Specimens From Patients With Thyroid Carcinoma. Arch. Pathol. Lab. Med..

[9] Vu-Phan D., Koenig R.J. (2014). Genetics and epigenetics of sporadic thyroid cancer. Mol. Cell. Endocrinol..

[10] Oishi N., Kondo T., Ebina A., Sato Y., Akaishi J., Hino R., Yamamoto N., Mochizuki K., Nakazawa T., Yokomichi H. (2017). Molecular alterations of coexisting thyroid papillary carcinoma and anaplastic carcinoma: Identification of TERT mutation as an independent risk factor for transformation. Mod. Pathol..

[11] Fleischhacker M., Schmidt B. (2007). Circulating nucleic acids (CNAs) and cancer—A survey. Biochim. Biophys. Acta BBA Rev. Cancer.

[12] Salvianti F., Giuliani C., Petrone L., Mancini I., Vezzosi V., Pupilli C., Pinzani P. (2017). Integrity and Quantity of Total Cell-Free DNA in the Diagnosis of Thyroid Cancer: Correlation with Cytological Classification. Int. J. Mol. Sci..

[13] Lupo M., Guttler R., Geck Z., Tonozzi T.R., Kammesheidt A., Braunstein G.D. (2018). Is Measurement of Circulating Tumor DNA of Diagnostic Use in Patients with Thyroid Nodules?. Endocr. Pract..

[14] Bettegowda C., Sausen M., Leary R.J., Kinde I., Wang Y., Agrawal N., Bartlett B.R., Wang H., Luber B., Alani R.M. (2014). Detection of circulating tumor DNA in early- and late-stage human malignancies. Sci. Transl. Med..

[15] Kim B.H., Kim I.J., Lee B.J., Lee J.C., Kim I.S., Kim S.J., Kim W.J., Jeon Y.K., Kim S.S., Kim Y.K. (2015). Detection of plasma BRAF(V600E) mutation is associated with lung metastasis in papillary thyroid carcinomas. Yonsei Med. J..

[16] Pupilli C., Pinzani P., Salvianti F., Fibbi B., Rossi M., Petrone L., Perigli G., De Feo M.L., Vezzosi V., Pazzagli M. (2013). Circulating BRAFV600E in the diagnosis and follow-up of differentiated papillary thyroid carcinoma. J. Clin. Endocrinol. Metab..

[17] Chen A.Y., Braunstein G.D., Anselmo M.S., Jaboni J.A., Viloria F.T., Neidich J.A., Li X., Kammesheidt A. (2017). Mutation detection with a liquid biopsy 96 mutation assay in cancer patients and healthy donors. Cancer Transl. Med..

[18] Condello V., Macerola E., Ugolini C., De Napoli L., Romei C., Materazzi G., Elisei R., Basolo F. (2018). Analysis of circulating tumor DNA does not improve the clinical management of patients with locally advanced and metastatic papillary thyroid carcinoma. Head Neck.

[19] Lubitz C.C., Parangi S., Holm T.M., Bernasconi M.J., Schalck A.P., Suh H., Economopoulos K.P., Gunda V., Donovan S.E., Sadow P.M. (2016). Detection of Circulating BRAF(V600E) in Patients with Papillary Thyroid Carcinoma. J. Mol. Diagn..

[20] Han J.Y., Choi J.J., Kim J.Y., Han Y.L., Lee G.K. (2016). PNA clamping-assisted fluorescence melting curve analysis for detecting EGFR and KRAS mutations in the circulating tumor DNA of patients with advanced non-small cell lung cancer. BMC Cancer.

[21] George J.R., Henderson Y.C., Williams M.D., Roberts D.B., Hei H., Lai S.Y., Clayman G.L. (2015). Association of TERT Promoter Mutation, But Not BRAF Mutation, With Increased Mortality in PTC. J. Clin. Endocrinol. Metab..

[22] Laurent-Puig P., Pekin D., Normand C., Kotsopoulos S.K., Nizard P., Perez-Toralla K., Rowell R., Olson J., Srinivasan P., Le Corre D. (2015). Clinical relevance of KRAS-mutated subclones detected with picodroplet digital PCR in advanced colorectal cancer treated with anti-EGFR therapy. Clin. Cancer Res..

[23] Kulemann B., Rosch S., Seifert S., Timme S., Bronsert P., Seifert G., Martini V., Kuvendjiska J., Glatz T., Hussung S. (2017). Pancreatic cancer: Circulating Tumor Cells and Primary Tumors show Heterogeneous KRAS Mutations. Sci. Rep..

[24] Hay I.D. (1990). Papillary thyroid carcinoma. Endocrinol. Metab. Clin. N. Am..

[25] Leboulleux S., Rubino C., Baudin E., Caillou B., Hartl D.M., Bidart J.M., Travagli J.P., Schlumberger M. (2005). Prognostic factors for persistent or recurrent disease of papillary thyroid carcinoma with neck lymph node metastases and/or tumor extension beyond the thyroid capsule at initial diagnosis. J. Clin. Endocrinol. Metab..

[26] Yang S., Che S.P., Kurywchak P., Tavormina J.L., Gansmo L.B., Correa de Sampaio P., Tachezy M., Bockhorn M., Gebauer F., Haltom A.R. (2017). Detection of mutant KRAS and TP53 DNA in circulating exosomes from healthy individuals and patients with pancreatic cancer. Cancer Biol. Ther..

[27] Gormally E., Vineis P., Matullo G., Veglia F., Caboux E., Le Roux E., Peluso M., Garte S., Guarrera S., Munnia A. (2006). TP53 and KRAS2 mutations in plasma DNA of healthy subjects and subsequent cancer occurrence: A prospective study. Cancer Res..

[28] Stanta G., Bonin S. (2018). Overview on Clinical Relevance of Intra-Tumor Heterogeneity. Front. Med..

[29] Tanzima Nuhat S., Sakata-Yanagimoto M., Komori D., Hattori K., Suehara Y., Fukumoto K., Fujisawa M., Kusakabe M., Matsue K., Wakamatsu H. (2018). Droplet digital polymerase chain reaction assay and peptide nucleic acid-locked nucleic acid clamp method for RHOA mutation detection in angioimmunoblastic T-cell lymphoma. Cancer Sci..

[30] Chan K.C., Yeung S.W., Lui W.B., Rainer T.H., Lo Y.M. (2005). Effects of preanalytical factors on the molecular size of cell-free DNA in blood. Clin. Chem..

[31] Nixon A.M., Provatopoulou X., Kalogera E., Zografos G.N., Gounaris A. (2017). Circulating thyroid cancer biomarkers: Current limitations and future prospects. Clin. Endocrinol..

